# Betriebliche Mobilitätsmanagementmaßnahmen – ein Anstoß für Veränderungen in Mobilitätsverhalten und -einstellungen Mitarbeitender?

**DOI:** 10.1007/s00548-021-00710-0

**Published:** 2021-06-09

**Authors:** Stefan Saake, Jörg Lahner, Eike Matthies

**Affiliations:** 1 Institut für Verkehrswesen, Fachgebiet Verkehrsplanung und Verkehrssysteme, Universität Kassel, Mönchebergstraße 7, 34125 Kassel, Deutschland; 2 Ghair of Economic Development and Gorparate Govemance, Faculty of Resource Management, Hochschule für angewandte Wissenschaft und Kunst, Büsgenweg 1a, 37077 Göttingen, Deutschland; 3 Department of Economics, Georg-August-University of Göttingen, Humboldtallee 3, 37073 Göttingen, Deutschland

**Keywords:** Betriebliches Mobilitätsmanagement, Verkehrssektor, Verhaltensänderung, Mobilitätsverhalten, Verkehrsmittelwahl, Corporate mobility management, Transport sector, Behaviour change, Mobility behaviour, Mode of transport

## Abstract

Betriebliches Mobilitätsmanagement (BMM) fokussiert auf die Verkehrsgestaltung hinsichtlich Pendel- und Berufsverkehr, Dienstreisen und Kundenverkehr. Ziel ist es, beispielsweise Parkplatzauslastungen zu verringern oder Verhaltensänderungen bei den Mitarbeitenden hinsichtlich der Verkehrsmittelwahl hervorzurufen. Bisher ist BMM in Deutschland wenig implementiert und in geringem Umfang erforscht.

Vor diesem Hintergrund stellt dieser Artikel das Instrument BMM theoretisch dar und präsentiert ein zugehöriges Praxisprojekt, in dem verschiedene Maßnahmen mit dem Ziel der Veränderung von Mobilitätsverhalten und -einstellungen durchgeführt wurden. Die Evaluation dieses Projektes erlaubt den Rückschluss, dass BMM und Veränderungen in den Einstellungen der Mitarbeiterinnen und Mitarbeiter korrelieren. Eine direkte Veränderung des tatsächlichen Verkehrsverhaltens konnte jedoch nicht nachgewiesen werden. Die Untersuchung basiert auf der statistischen Analyse von 2 Befragungen aus den Jahren 2019 und 2020. Die Darstellung der Ergebnisse wird von Policy Implications abgerundet. In theoretischer Hinsicht basieren die Auswertungen dieses Artikels auf der Annahme, dass psychologische Einflussfaktoren das Mobilitätsverhalten beeinflussen.

## Abstract (en)

Corporate mobility management (CMM) focuses on transport design concerning commuting, business trips and client transport. It is aimed, for example, at reducing occupancy rates of car parks or changing the mobility behaviour of employees with regard to their choice of transport. Until now, CMM has been implemented and researched very little in Germany.

Against this background, this article depicts the instrument of CMM theoretically and presents a related practical project in which various measures were implemented with the aim of changing mobility behaviour and attitudes. The evaluation of this project allows the conclusion to be drawn that CMM correlates with changes in the attitudes of employees. However, a direct change in actual traffic behaviour could not be proven. The study is based on the statistical analysis of two surveys conducted in 2019 and 2020. The presentation of the results rounds off with policy implications. In theoretical terms, the analyses in this article are based on the assumption that psychological factors have a decisive influence on mobility behaviour.

## Verkehrsgestaltung durch betriebliches Mobilitätsmanagement

In Deutschland wurde das Konzept des Mobilitätsmanagements 1995 durch die Forschungsgesellschaft für Straßen- und Verkehrswesen (FGSV) eingeführt (FGSV 1995). Mobilitätsmanagement wurde bei der Einführung „erstmals als komplementäres Planungsfeld zum klassischen Verkehrsmanagement betrachtet“ (Schwedes et al. 2017, S. 9). Ziel war eine Verlagerung und Reduktion des Verkehrs. Seit der Einführung hat sich ein Verständnis von Mobilitätsmanagement als systematischer Ansatz herauskristallisiert (Schwedes et al. 2017).

Das Betriebliche Mobilitätsmanagement (BMM) fokussiert auf Verkehrsgestaltung hinsichtlich Pendel- und Berufsverkehr, Dienstreisen und -wegen sowie Kunden- und Besucherverkehr (Institut für Landes- und Stadtentwicklungsforschung des Landes Nordrhein-Westfalen und [ILS] 2012). Es definiert damit den Betrieb als Quell- und als Zielort von Verkehrsströmen (ILS 2001). Mit Maßnahmen aus dem BMM wird darauf abgezielt, Parkplatzauslastungen zu verringern und Flächenversiegelung durch zusätzlichen Parkraum zu vermeiden, Verhaltensänderungen bei den Mitarbeiten hinsichtlich Verkehrsmittelwahl und damit verknüpft die Gesundheit der Mitarbeitenden zu fördern, das Betriebsimage zu verbessern und Treibhausgas(THG)-Emissionen zu reduzieren (Schuppan 2020).

Das Konzept des BMM ist in Deutschland bisher kaum etabliert und weiterentwickelt worden (Schwedes et al. 2017). Der Forschungsstand ist fragmentiert (Schwedes et al. 2016). Hier knüpft dieser Beitrag an.

Um die Potenziale des BMM zu identifizieren und seinen Beitrag zu Veränderungen in Mobilitätsverhalten und -einstellungen Mitarbeitender abschätzen zu können, ist die Evaluation von erprobten Einzelmaßnahmen und/oder Maßnahmenbündeln notwendig. Daher werden in diesem Artikel die Evaluationsergebnisse eines BMM-Praxisprojektes dargestellt. Es wird die verkehrliche Wirkung eines Maßnahmenbündels untersucht und überprüft, ob und in welchem Ausmaß im Rahmen von BMM Einstellungsveränderungen innerhalb der Belegschaft und ein verändertes Mobilitätsverhalten erreichbar sind. Diese Ausarbeitung leistet damit einen Beitrag zur Erforschung des BMM-Potenzials hinsichtlich Verhaltensänderungen und der Veränderung von Einstellungen.

## Stand der Forschung

### Betriebliches Mobilitätsmanagement

Die FGSV definiert Mobilitätsmanagement als „die vorausschauende systematische Vorbereitung, Umsetzung und (Wirkungs‑)Kontrolle von Entscheidungen, die das Mobilitätsverhalten von Personen durch informatorische, kommunikative, organisatorische, normative sowie auch bauliche und betriebliche Maßnahmen in Richtung ökologischer, ökonomischer und sozialer Nachhaltigkeit beeinflussen sollen“ (Blees 2016, S. 13).

Das BMM wendet dieses Planungsinstrument auf der Unternehmensebene an. Zentral ist die Gestaltung „des von einem Betrieb erzeugten Verkehrs“ (Witte 2015, S. 7). Mobilitätsgewohnheiten der Mitarbeitenden sollen gelenkt und die Verkehrsmittelwahl soll zugunsten nachhaltiger Transportmittel beeinflusst werden (Witte 2015). Neben infrastrukturellen Maßnahmen liegt der Fokus vor allem auf nichtinvestiven Ansätzen (bspw. Kommunikationsmaßnahmen) (DifU 2010). Trotz seines Potenzials (Guntermann et al. 2014) spielt BMM bisher nur eine untergeordnete Rolle (Schwedes et al. 2017).

### Verhaltensänderungen im Mobilitätsbereich

Um eine Verhaltensänderung hervorzurufen, ist es hilfreich, das Mobilitätsverhalten der Zielgruppe zu verstehen. Im Hinblick auf die Beeinflussung des Mobilitätsverhaltens werden harte Faktoren, wie z. B. infrastrukturelle Ausstattung, und weiche bzw. individuelle Faktoren, die nicht objektivierbar und oft psychologischer Natur sind, unterschieden. Der nachfolgende Überblick veranschaulicht, dass weiche Faktoren, beispielsweise (kulturelle) Normen (Schwedes und Rammert 2020), immer häufiger zur Erklärung herangezogen werden.

Einen großen Einfluss auf die Erforschung des Mobilitätsverhaltens hat die Theorie des geplanten Verhaltens (Theory of Planned Behavior [TOPB]) von I. Ajzen (Ajzen 1991). Ajzen untersucht, welche Faktoren für eine Verhaltensänderung entscheidend sind. Die Grundannahme seiner Arbeit ist, dass psychologische Einflussfaktoren zentral sind, um eine Verhaltensänderung zu bewirken. Die TOPB stellt bis heute die Grundlage von einer Vielzahl an Forschungsarbeiten dar (Yuriev et al. 2020).

Hunecke (2015) bezieht bei der Untersuchung von Änderungen im Mobilitätsverhalten ebenfalls psychologische Einflussfaktoren (z. B. Werte und Normen) ein. Fastenmeier und Gstalter (2011) folgend ist hinzuzufügen, dass die kognitive und die emotionale Komponente der Einstellung zu verschiedenen Verkehrsmitteln wichtig sind. Wissen und Umweltbewusstsein allein haben einen geringen direkten Zusammenhang zum tatsächlichen Verkehrsmittelwahlverhalten. Sie dienen eher als Katalysatoren zur Akzeptanzbildung.

Insgesamt erklärt das Gros der hier vorgestellten Ansätze das Mobilitätsverhalten unter Einbeziehung von psychologischen Überlegungen. Die Relevanz harter Faktoren, wie z. B. eine vorhandene (Fahrrad‑)Verkehrsinfrastruktur oder akzeptable Weglängen, darf jedoch in einer ganzheitlichen Betrachtung keinesfalls unberücksichtigt bleiben. Nicht unerwähnt bleiben sollte auch die Bedeutung von Routinen und Lebenslaufereignissen, auf die in diesem Artikel nicht näher eingegangen wird (bspw. Scheiner 2009; Müggenberg 2017).

Die nachfolgenden Ausführungen zu dem Praxisprojekt MAXIH veranschaulichen, inwiefern Änderungen in der Einstellung von Mitarbeitenden nachgewiesen werden können, wie diese durch BMM gefördert werden und ob sie mit tatsächlichen Verhaltensänderungen hinsichtlich Verkehrsmittelnutzung auf Arbeits- und Freizeitwegen und mit Veränderungen in den Einstellungen zu Verkehrsmitteln einhergehen.

## Datenerhebung und Forschungsdesign

### Das Praxisprojekt

Das Projekt „Mobil, innovativ, nachhaltig. Der Landkreis Holzminden gibt Gas – Maximale PS für den Klimaschutz“, kurz MAXIH, wurde durchgeführt, um die betriebliche Mobilität der Kreisverwaltung Holzminden nachhaltiger zu gestalten und Verhaltensänderungen im Mobilitätsverhalten der Mitarbeitenden zu induzieren. Die Kreisverwaltung beschäftigte zum Stichtag 31.07.2019 insgesamt 616 Personen.

**Abb. 1:**
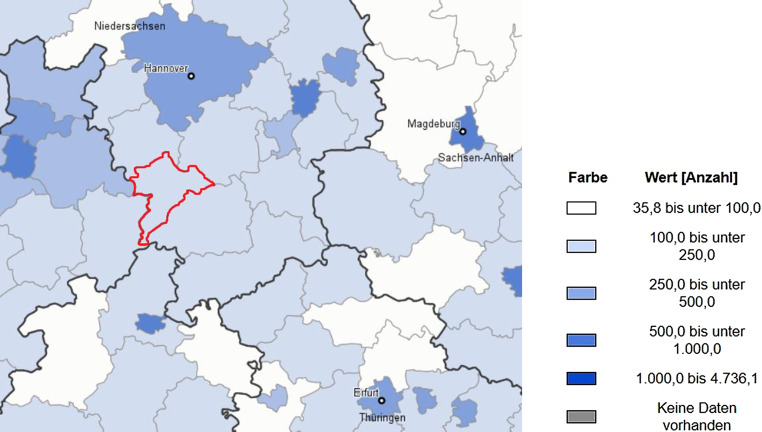
Der Landkreis Holzminden im südlichen Niedersachsen. (Quelle: Regionalatlas Deutschland. Statistische Ämter des Bundes und der Länder, Deutschland, 2020); Indikatoren des Themenbereichs „Bevölkerung“; Bevölkerungsdichte (Einwohner je qkm); Jahr 2018, Kreise und kreisfreie Städte; 5 Klassen, manuelle Klassifizierung

Der Landkreis Holzminden liegt im südlichen Niedersachsen (siehe Abb. 1, rot markiert) und ist ländlich geprägt. Der motorisierte Individualverkehr besitzt daher einen hohen Stellenwert (Kuhnimhof und Nobis 2018).

Im Projekt MAXIH wurden 10 Maßnahmen umgesetzt (siehe Tab. 1), die das Mobilitätsverhalten der Mitarbeitenden und die betriebliche Mobilität der Kreisverwaltung umwelt- und klimafreundlicher gestalten sollten.

**Tab. 1:** Maßnahmen im Projekt MAXIH

Nr.	Maßnahme	Beschreibung	Zielbereiche
1	Anschaffung von Pedelecs, Fahrrädern und Lastenrad für Dienstwege	Reduktion von THG-Emissionen, Kostenvermeidung, Steigerung der Gesundheit der Mitarbeitenden	Dienstreisen
2	Information und Kommunikation zu klimafreundlicher Mobilität – Mobilitätstag	Vermittlung von Informationen zum sicheren Radfahren und zur Anreise mit ÖPNV. Schulung der Mitarbeitenden zum spritsparenden und sicheren Fahren	Kommunikation
3	Information und Kommunikation zu klimafreundlicher Mobilität – Wettbewerb	Aufmerksamkeit für die Thematik, Anreize zur Fahrradnutzung auf dem Arbeitsweg	Kommunikation
4	Anschaffung umweltfreundlicher Dienstwagen	Anschaffung eines Elektroautos. Ersetzen von 2 Pkws der Dienstwagenflotte durch Compressed natural gas CNG-Pkws	Dienstreisen
5	Ausstattung der Dienstwagen mit Navigationsgeräten	Ausstattung mit Navigationsgeräten, um Kosten für Umwege und das Stresslevel der Mitarbeitenden zu reduzieren	Dienstreisen
6	Training zum Fahren mit Elektroautos	Mit Schulungsprogrammen für richtiges elektrisches Fahren Vorurteilen und Wissensdefiziten begegnen, die Sicherheit im Umgang steigern	Dienstreisen
7	Förderung von Fahrgemeinschaften durch eine Mitfahrzentrale	Einrichtung einer Mitfahrzentrale zur Förderung von Fahrgemeinschaften	Pendelwege
8	Planungstool Dienstreisen	Automatisierte Vergabe der Fahrzeuge einschl. Fahrrädern	Dienstreisen
9	Machbarkeitsstudie Fahrradverleihsystem für Bevölkerung in Region Holzminden – Höxter	Etablierung eines bundesländerübergreifenden FVS	Regionales Mobilitätsangebot
10	Entwicklung Umsetzungskonzept FVS	Nach Prüfung der Machbarkeit eines FVS sollte – bei positivem Ergebnis – das Umsetzungskonzept entwickelt werden	Regionales Mobilitätsangebot

*THG* Treibhausgase, *ÖPNV* Öffentlicher Personennahverkehr, *CNG* „compressed natural gas“, *FVS* Fahrradverleihsystem

### Aufbau der Befragung

Im Rahmen der wissenschaftlichen Evaluation wurden 2 Onlinebefragungen der Mitarbeiterinnen und Mitarbeiter der Kreisverwaltung durchgeführt. Das Verkehrsverhalten der Belegschaft sollte untersucht und die Einstellungen zu verschiedenen Themen, die für das Verkehrsverhalten mutmaßlich relevant sind, sollten erfragt werden.

Es wurden im Abstand von einem Jahr, im Mai 2019 und 2020, 2 Erhebungen durchgeführt. Alle Mitarbeiterinnen und Mitarbeiter der Kreisverwaltung wurden per E‑Mail kontaktiert und erhielten einen Link zum Onlinefragebogen. Im Jahr 2019 konnten 80, im Jahr 2020 94 vollständige Fragebögen ausgewertet werden. Dies entspricht einer Rücklaufquote von 13 bzw. 15 %.

Die Angaben zum Verkehrsverhalten wurden so abgefragt, dass die antwortende Person eine Matrix vervollständigte, in welcher sie angab, an wie vielen Tagen sie verschiedene Verkehrsmittel für verschiedene Wegzwecke nutzte (von Behren et al. 2017). Um die Einstellung der Mitarbeiterinnen und Mitarbeiter zu ermitteln, wurde ein Set von 27 Fragen genutzt (Hunecke 2015; von Behren et al. 2017). Verteilt auf 10 Themenbereiche wurden Fragen auf einer 5‑stufigen Likert-Skala abgefragt.

Es wurde keine Panelerhebung durchgeführt, aber die vorgefundenen demografischen Angaben zu Alter, Haushaltsgröße und Geschlecht weisen keine signifikanten Unterschiede auf. Die Datensätze sind miteinander vergleichbar.

## Ergebnisse

Nachfolgend wird den Fragen nachgegangen, ob und in welchem Ausmaß eine Veränderung des Verkehrsverhaltens innerhalb der Belegschaft nachzuweisen ist und ob Einstellungsveränderungen in Bezug auf das Mobilitätsverhalten zu finden sind.

### Änderungen in der Häufigkeit der Verkehrsmittelnutzung

Lediglich bei der Nutzung des Autos für Freizeitwege konnte bei einem Konfidenzniveau von 95 % eine statistisch signifikante Verringerung der Nutzungshäufigkeit nachgewiesen werden. Während das Auto im Jahr 2019 im Schnitt an 2,39 Tagen genutzt wurde, sank der Wert im Jahr 2020 auf 1,71. Die geringen Auswirkungen auf Pendelwege sind möglicherweise darauf zurückzuführen, dass nur eine Projektmaßnahme direkt darauf fokussierte. Auch ist das Mobilitätsverhalten in ländlichen Räumen durch die geographischen Bedingungen und wenige Pkw-Alternativen geprägt (Kuhnimhof und Nobis 2018; Reichert-Schick 2015). Wissen und Umweltbewusstsein – der Schwerpunkt der hier untersuchten Maßnahmen – haben dahingegen einen eher geringen direkten Zusammenhang zur tatsächlichen Verkehrsmittelwahl.

### Änderungen von mobilitätsbezogenen Einstellungen

Die beobachteten Einstellungsveränderungen sind in Tab. 2 dargestellt. Der höchste Zustimmungswert auf der Skala ist eine 5, der niedrigste eine 1.

**Tab. 2:** Einstellungsfragen im Vergleich

*Themenfeld* (Fragetext)	Zustimmung nach Jahr (arithm. Mittel auf 5‑stufiger Skala)	
	2019 (*N* = 80)	2020 (*N* = 94)
Empfundene Norm 1* (Menschen, die mir wichtig sind, finden es gut, wenn ich für alltägliche Dinge den ÖPNV anstelle eines Autos benutze.)	2,49	2,85
Empfundene Norm 2** (Leute, die mir wichtig sind, denken, dass ich den ÖPNV anstelle eines Autos benutzen sollte.)	2,04	2,34
Persönliche Norm 1** (Aufgrund meiner Prinzipien fühle ich mich persönlich verpflichtet, für alltägliche Dinge umweltfreundliche Verkehrsmittel zu nutzen.)	3,04	3,40
Persönliche Norm 2** (Ich fühle mich verpflichtet, durch meine Wahl des Transportmittels einen Beitrag zum Klimaschutz zu leisten.)	3,27	3,58

^*^Signifikante Unterschiede bei Konfidenzniveau von 90 %, ^**^signifikante Unterschiede bei Konfidenzniveau von 95 %

Bei einem Konfidenzniveau von 95 % sind die Veränderungen bei den Themenfeldern „Empfundene Norm 2“ sowie „Persönliche Norm 1“ und „Persönliche Norm 2“ signifikant. Bei einem Konfidenzniveau von 90 % sind auch die Veränderungen bei „Empfundene Norm 1“ signifikant. Somit wird belegt, dass während der Projektlaufzeit eine Veränderung des Normempfindens innerhalb der Belegschaft hinsichtlich verkehrs- und klimarelevanter Themen erfolgte.

Die Maßnahmen der Kreisverwaltung könnten Veränderungsprozesse bei den Mitarbeiterinnen und Mitarbeitern in Gang gesetzt haben. Ein Bewusstsein für eine klimafreundlichere Mobilität konnte geschaffen werden. Gerade im Hinblick auf persönliche Einstellungen (persönliche Norm) und empfundene Norm können sich die BMM-Maßnahmen ausgewirkt haben, da über Information und Kommunikation im Rahmen der einzelnen Maßnahmen Wissen zu nachhaltiger Mobilität im Betrieb vermittelt wurde. Dies gilt für die individuellen Angestellten und damit den Einfluss auf ihre persönliche Norm sowie für die Kolleginnen und Kollegen, also das Umfeld, und damit die empfundene Norm.

## Fazit

Betriebliches Mobilitätsmanagement hat das Potenzial, Verhaltensänderungen hervorzurufen und die Einstellungen der Mitarbeiterinnen und Mitarbeiter zu beeinflussen. Die Größe dieses Beitrages zur tatsächlichen Verkehrsmittelwahl sollte, zumindest im hier vorgestellten Projektkontext, jedoch nicht überschätzt werden. Signifikante Veränderungen wurden bei den persönlichen und empfundenen Normen festgestellt. Folglich wurde das Bewusstsein der Mitarbeiterinnen und Mitarbeiter, dass die persönliche Mobilität einen Beitrag zum Klima- und Umweltschutz leisten kann und muss, geschärft. Diese Bewusstseinsänderung kann einen ersten Schritt hin zu einer tatsächlichen Verhaltensänderung darstellen. Für deren Realisierung ist es jedoch unverzichtbar, dass alternative Mobilitätsformen angeboten werden.

**Abb. 2:**
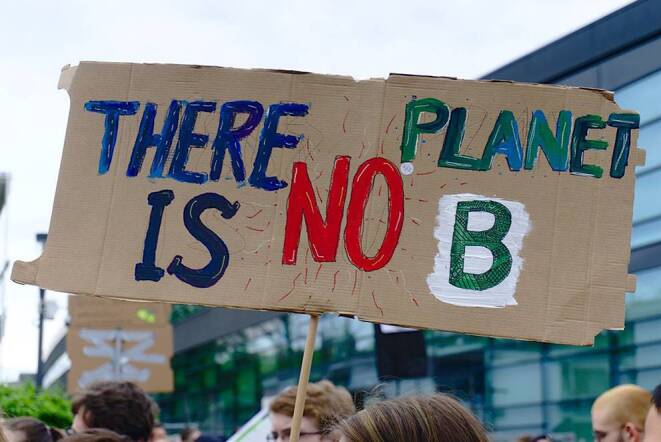
Klimaschutzproteste im Rahmen von Fridays for Future. (Quelle: pixabay.com/NiklasPntk)

Eine Unsicherheit in der Auswertung besteht hinsichtlich der Auswirkungen der COVID-19-Pandemie auf das Verkehrsverhalten. Es liegt nahe, dass die Erfahrungen aus dem April 2020 Auswirkungen auf die Beantwortung der Befragung im Mai 2020 hatten. Auch sollte der gesamtgesellschaftliche Kontext nicht ausgeklammert werden. Es kann nicht beurteilt werden, welcher Anteil an den vorgefundenen Einstellungsveränderungen Klimaschutzbewegungen wie etwa Fridays for Future zuzurechnen ist, die, wie beispielhaft in Abb. 2 dargestellt, die öffentliche Wahrnehmung für das Thema Klimaschutz schärfen. Daher ist der kausale Effekt von BMM auf die Einstellungen der Mitarbeiterinnen und Mitarbeiter nicht klar. Hier gibt es weiteren Forschungsbedarf.
